# Dynamics of Multisystem Inflammatory Syndrome in Children (MIS-C) associated to COVID-19: steady severity despite declining cases and new SARS-CoV-2 variants—a single-center cohort study

**DOI:** 10.1007/s00431-025-06153-1

**Published:** 2025-05-07

**Authors:** Thomas Carzaniga, Valeria Calcaterra, Luca Casiraghi, Tommaso Inzani, Stephana Carelli, Gabriele Del Castillo, Danilo Cereda, Gianvincenzo Zuccotti, Marco Buscaglia

**Affiliations:** 1 Department of Medical Biotechnology and Translational Medicine, University of Milan, Segrate, 20054 Italy; 2 Department of Pediatrics, Buzzi Children’s Hospital, Milano, 20154 Italy; 3 Pediatrics and Adolescentology Unit, Department of Internal Medicine, University of Pavia, Pavia, 27100 Italy; 4 Pediatric Clinical Research Center “Romeo ed Enrica Invernizzi,” Department of Biomedical and Clinical Science, University of Milan, Milan, Italy; 5 Center of Functional Genomics and Rare Diseases, Buzzi Children’s Hospital, Milan, 20154 Italy; 6 Prevention Operational Unit, General Directorate of Welfare, Lombardy Region, Milan, Italy; 7 Department of Biomedical and Clinical Sciences, L. Sacco, University of Milan, Milan, 20157 Italy

**Keywords:** Multisystem inflammatory syndrome, Children, SARS-CoV-2, Infection, Variants of concern, Serum antibody fingerprint, Label-free microarray biosensor

## Abstract

Multisystem Inflammatory Syndrome in Children (MIS-C) is a serious condition associated with SARS-CoV-2 infection. The relationship between SARS-CoV-2 variants of concern (VOCs) and the occurrence and severity of MIS-C is unknown. We analyzed the dynamics of MIS-C in the Milan metropolitan area (Italy) during the COVID-19 pandemic, focusing on the epidemiologic trends and disease severity in relation to different VOCs in a single-center study. Fifty-seven MIS-C patients (mean 8.3 ± 3.8 years) admitted to the Pediatric Department of Buzzi Children’s Hospital in Milan, Italy, between November 2020 and July 2022, were retrospectively included in the study. The SARS-CoV-2 variant was retrospectively identified from serological fingerprinting (profiles of serum antibodies targeting different variants of SARS-CoV-2 obtained by a label-free microarray biosensor) or by the variant of prevalence. Two main periods of MIS-C case accumulation were observed. The peak of MIS-C cases rate in December 2020 reached 0.6 cases per day, which is nearly double the rate observed in February 2022, despite the larger number of infected subjects. Although the WT variant exhibited a broader range of severity score values, the score distributions for the different variants do not show statistically relevant differences. *Conclusion*: The results clearly show a decrease in the incidence of MIS-C in relation to infections, but also support the concept that severity of MIS-C remained essentially unchanged across different virus variants, including Omicron. The course of MIS-C, once initiated, is independent from the characteristics of the triggering variants, although later variants may be considered less likely to induce MIS-C.

**Table:** 

What is known: • MIS-C is a rare systemic inflammatory disorder that arises as a post-infectious complication temporally related to SARS-CoV-2 infection. • Fluctuations in MIS-C incidence were observed throughout the pandemic, with the latest variants associated with a lower incidence.		
What is new: • The SARS-CoV-2 variant of infection can be retrospectively confirmed by serum antibody fingerprinting using a label-free microarray biosensor. • Despite the decreasing incidence, MIS-C severity has remained essentially unchanged across SARS-CoV-2 variants.

**Supplementary Information:**

The online version contains supplementary material available at 10.1007/s00431-025-06153-1.

## Introduction

Multisystem Inflammatory Syndrome in Children (MIS-C) is a rare but serious condition associated with SARS-CoV-2 infection. Among children with confirmed exposure to COVID-19, less than 1% developed MIS-C [1]. Epidemiological characteristics include a higher prevalence in males, a peak incidence between 6 and 12 years of age, and distinct racial or ethnic predispositions, with Hispanic and non-Hispanic Black individuals being at the highest risk [2–4].

The pathogenic mechanism of MIS-C remains uncertain, though the most widely accepted theory involves a cytokine storm and the role of adaptive immunity following SARS-CoV-2 infection. Additionally, certain intrinsic susceptibility factors in the host have been identified, and molecular mimicry has also been suggested as part of the disease’s pathogenesis [5, 6]. MIS-C typically occurs a few weeks after a child has been infected with the virus, involving inflammation across multiple organ systems. The most common manifestations involve the gastrointestinal tract, cardiovascular system, hematological system, and mucocutaneous system. The respiratory tract, neurological system, musculoskeletal system, and kidneys are less frequently affected. Most patients are previously healthy children without significant comorbidities, aside from asthma and obesity [7].

Since late 2020, several SARS-CoV-2 variants of concern (VOCs), including Alpha, Beta, Gamma, Delta, and Omicron, have emerged, significantly influencing the spread and severity of COVID-19. Each VOC carries mutations that can affect the virus's transmissibility, severity of illness, and ability to evade immune responses from either natural infection or vaccination [8, 9]. With the emergence of SARS-CoV-2 VOCs, interest has grown in understanding the relationship between these variants and the occurrence of MIS-C. Some studies have highlighted differences in MIS-C incidence among patients infected by various SARS-CoV-2 variants. Notably, MIS-C incidence was lower during the Omicron wave compared to the Delta wave, with Omicron-related cases perceived as generally milder than those seen in the periods of the previous variants [10–12]. However, it remains unclear whether VOCs directly influence MIS-C occurrence or if the increase in MIS-C cases is merely a reflection of the overall rise in infections driven by these variants. Moreover, fluctuations in MIS-C incidence throughout the pandemic may also be attributed to a combination of various factors, including rising seroprevalence due to previous infections and/or vaccination [13].

The aim of this study was to analyze the dynamics of MIS-C in the metropolitan area of the city of Milan (Italy) during the COVID-19 pandemic, focusing on the epidemiologic trends and disease severity in relation to different VOCs, both before and after the availability of vaccinations for children.

## Methods

### Subjects with MIS-C

This retrospective study included 57 children admitted to the Pediatric Department of Buzzi Children’s Hospital in Milan, Lombardy region, Italy, between November 2020 and July 2022 who were diagnosed with MIS-C according to CDC criteria [14]. The hospital is a regional center of reference for the diagnosis and treatment of MIS-C during pandemic. To assess multisystem involvement severity, specific scores (0–2 points) were assigned to multiple clinical domains: renal, cardiac, gastrointestinal, neurological, pulmonary, dermatological/mucosal, endocrine, metabolic, and electrolyte disturbances. Summing these domain scores provides an overall severity measure. Additionally, key clinical outcomes, including ICU admission requirement, hospitalization duration, and fever occurrence, were also considered. Criteria details are in Supplementary Note S1 [15]. Demographic and clinical data, as well as vaccination status, were recorded for all patients. The study adhered to the Declaration of Helsinki and received approval from the institutional ethics committee (MI-1, n. 0034170; protocol number 2021/ST/138). Written consent was obtained from the patients’ guardians after explaining the study’s objectives.

### Auxological parameters

Physical assessments of children included evaluations of weight, height, BMI (Table 1). Height was measured with the patient standing barefoot using a Harpenden stadiometer, providing an accuracy of approximately ± 1 mm. Weight was recorded with the patient in underwear, standing upright on a platform scale, with a precision of about ± 100 g [16, 17]. BMI was calculated by dividing body weight (in kilograms) by the square of height (in meters), and BMI *z*-scores were derived using WHO reference standards [18]. The SARS-CoV-2 variant that caused the infection indicated in Table 1 was retrospectively identified from serological fingerprinting or by the variant of prevalence, as described in the “Results” section.

**Table 1:** Patient characteristics at admission

Characteristics	Total	Variant				
		**WT**	**Alpha**	**Delta**	**Omicron**	
					**Unvaccinated**	**Vaccinated**
**No. of patients**	57	27	6	12	8	4
**Males** (%) **Female** (%)	75 25	82 18	100 0	58 42	75 25	50 50
**Age** (years) **avg** **min/max**	8.3 ± 3.8 2/16	8.2 ± 3.9 2/15	8.0 ± 4.7 2/14	7.5 ± 3.8 3/16	9.0 ± 3.5 5/16	10.8 ± 2.1 8/13
**BMI** (kg/m^2^) **avg** **min/max**	18.1 ± 3.6 12.8/28.4	18.5 ± 3.9 13.5/28.4	17.1 ± 2.8 15.1/22.3	17.7 ± 3.7 12.8/23.9	18.6 ± 3.5 14.4/23.7	17.2 ± 3.0 13.2/20.1
**BMI z-score avg** **min/max**	0.14 ± 1.2 − 2.6/2.6	0.02 ± 1.1 − 2.2/1.9	− 0.17 ± 0.8 − 1.1/0.7	0.35 ± 1.5 − 2.4/2.3	0.68 ± 1.0 − 0.8/2.6	− 0.26 ± 1.9 − 2.6/1.5
**Severity score avg** **min/max**	12.2 ± 3.3 3/21	12.5 ± 3.9 3/21	11.0 ± 1.8 9/13	11 ± 3.2 6/17	12.9 ± 2.9 9/17	13.5 ± 1.3 12/15

### Samples and reagents

Plasma samples were obtained from patients at the Pediatric Department, Buzzi Children’s Hospital, Milan. All receptor-binding domains (RBD) of SARS-CoV-2 spike proteins (WT-RBD, α-RBD, γ-RBD, δ-RBD, o-RBD, o_1_-RBD, o_2_-RBD, and o_4/5_-RBD) obtained from HEK293 human embryonic kidney immortalized cell line were purchased from Sino Biological (Beijing, China). Nucleocapsid protein and Rabbit polyclonal antibody anti-human IgG were obtained from Abcam (Cambridge, UK; product code ab273530 and ab7155). Trimeric spike protein HexaPro was donated by Anton Schmitz and Günter Mayer [19, 20]. All the buffers and reagents were purchased from Merck (Darmstadt, Germany) and prepared using Milli-Q pure water.

### Serum antibody fingerprints

The profiles of serum antibody targeting different variants of SARS-CoV-2 were obtained by label-free microarray biosensor as described in [21]. Briefly, antigen proteins and control antibodies were covalently immobilized onto the surface of Reflective Phantom Interface (RPI) glass sensing chips in different spots forming a microarray [22]. After ozone cleaning, the chips were dip-coated with a copolymer of dimethylacrylamide (MCP2, Lucidant Polymers Inc., Sunnyvale, CA, USA) [20], and the arrays of spots were produced by an automated, non-contact dispensing system (sciFLEXARRAYER S3; Scienion AG, Berlin, Germany). Sample spikes were performed by adding 13 μL of patients’ plasma with a micropipette into disposable cartridges hosting the sensing chip, previously filled with 1.3 mL of measuring buffer (PBS 1 ×, pH 7.4, SDS 0.02% and sodium azide 0.02%). The cartridges were kept at 25 °C during the measurement through a thermalized holder, and rapid mixing of the solution was provided by magnetic stirring. A custom optical apparatus acquired time sequences of images of the spotted surface of the sensing chips. The amount of surface immobilized antigens was obtained from the brightness of each spot before the injection of the sample. Similarly, the amount of antibodies binding the surface antigens was obtained from the increase with time of the brightness of each spot after the sample injection. Each data point is obtained by averaging the signal from at least five spots with identical composition. For each antigen, the growth unit GU was obtained from the initial slope of the binding curves divided by the amount of surface antigens, and the relative GU (RGU) was computed as the ratio between the GU of each variant and that of WT variant.

### Statistical methods

Statistical analysis was made by using GraphPad Prism version 10. The possible influence of children’s age on the severity score was assessed by analyzing the dependence between these two parameters using linear regression (Supplementary Note S2). The possible influence of sex was assessed using Student’s *t*-test on the score distributions for males and females (Supplementary Note S3). The latency between MIS-C cases and COVID-19 infections was obtained from the analysis of the linear regression between the two sets of data with variable time delay (Supplementary Note S4). The uncertainties of the cumulative numbers *n* of MIS-C cases, infections, and vaccinations were obtained as 1.96 $\sqrt{n}$, representing 95% confidence interval for a Poisson distribution of events. The distributions of severity score values for the different SARS-CoV-2 variants were compared by using one-way analysis of variance (ANOVA) with Tukey’s post hoc analysis (Supplementary Note S5). All tests with P values > 0.3 were not considered statistically significant.

## Results

### The number of MIS-C cases showed two main peaks following corresponding peaks of infections

**Fig. 1:**
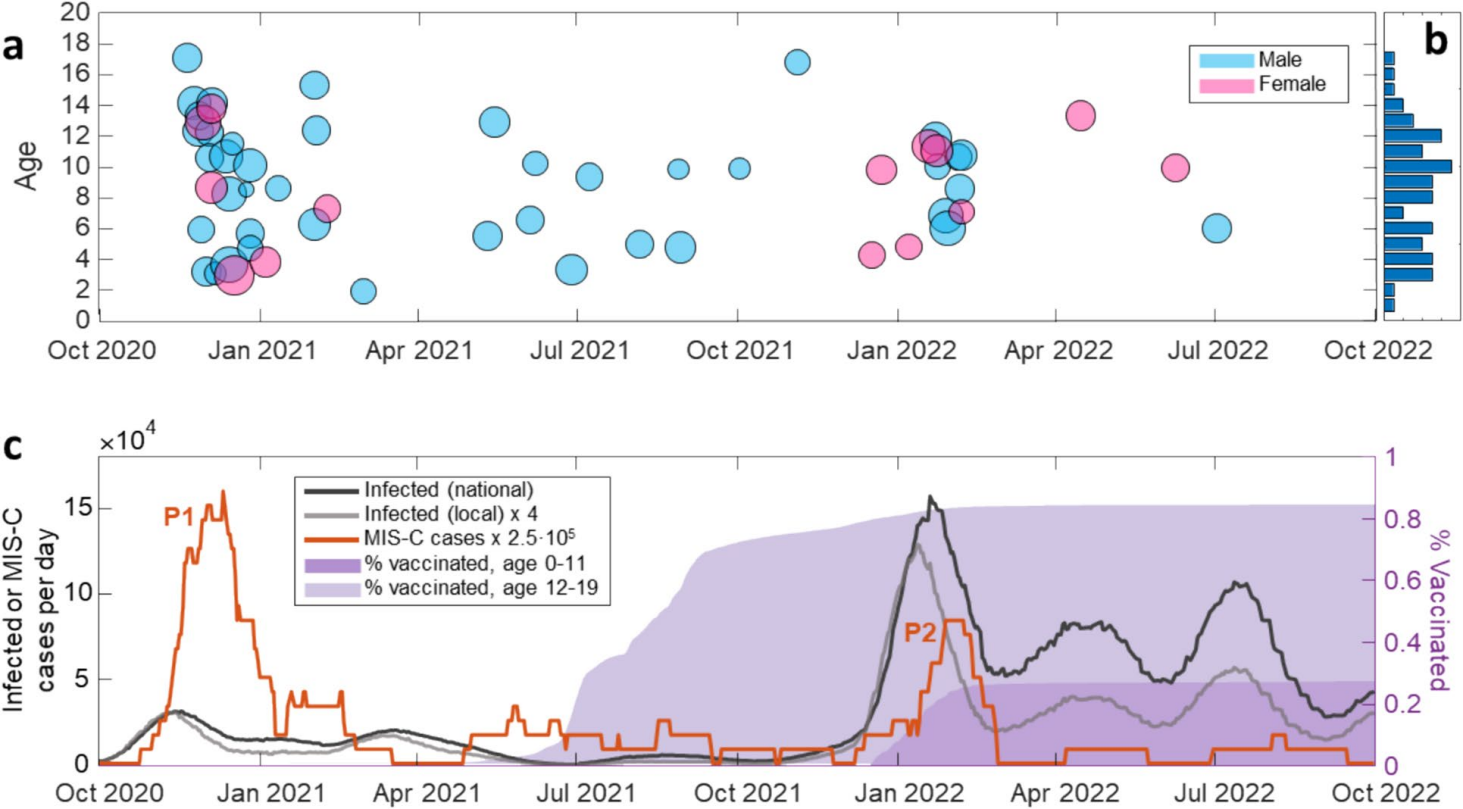
Dynamics of MIS-C cases and SARS-CoV-2 infection. **a** Time distribution of MIS-C cases diagnosed at Pediatric Department of Buzzi Children’s Hospital in Milan, Italy. For each data point, the horizontal axis indicates the initial time of hospitalization, the vertical axis reports the age of the child, the color of the circle represents the sex (cyan for males and magenta for females), and the circle size indicates the severity score [15] assigned to each case. **b** Age distribution of MIS-C cases of panel a. Each abscissa tick represents 2 cases. **c** Time dependence of MIS-C cases reported in panel **a** (orange), and of SARS-CoV-2 infections in Italy (black) [23] and in Lombardy region (grey) [24], computed as 30-day moving averages of daily data. The data of MIS-C cases are multiplied by a factor 2.5 × 10^5^ and the data of the number of infection in Lombardy are multiplied by a factor 4 to facilitate the comparison. The two peaks of MIS-C cases of December 2020 and February 2022 are indicated as P1 and P2, respectively. The shadow curves represent the percentage of vaccinated children in Lombardy with ages 0–11 (dark violet) and 12–19 (light violet)

In the two-year period covered by this study, from November 2020 up to July 2022, 57 children with age up to 17-year-old were diagnosed with MIS-C after admission at the Pediatric Department of Buzzi Children’s Hospital in Milan, Italy. No pre-existing illnesses or known comorbid conditions were found among the patients. A summary of the main characteristics at admission for the cohort of subjects considered in this study is presented in Table 1. We analyzed the temporal distribution of the MIS-C cases in relation to the characteristics of the subjects, of the infection and of the severity of the disease. Figure 1a reports the age and the date of hospital admission for each subject, as well as the sex (color of the points) and the severity of MIS-C (size of the points) quantified by the score proposed in [15]. The age distribution of the subjects (Fig. 1b) is rather uniform between 3 and 12 years, whereas fewer cases have been reported outside this range of age. Among the subjects with MIS-C, the number of males (43) is considerably larger, in agreement with previous studies [2–4]. Despite the effect of age and sex on the number of cases, the severity score does not display significant dependences on these factors (see Methods and Supplementary Notes S2 and S3). Figure 1a shows two main periods with accumulation of MIS-C cases: in December 2020 (P1) and February 2022 (P2). Figure 1c (orange line) reports the temporal dependence of the number of MIS-C cases per day computed by 30-day moving average. As expected from the data of Fig. 1a, two peaks emerge in the period P1 and P2. Figure 1c also reports the infection rate with SARS-CoV-2 virus over the Italian population (black line) [23] and the population of Lombardy (grey line) [24]. Beside a scaling factor due to the different size of the basins, the time behavior of the infections is quite similar for the two populations. The peaks of MIS-C follow by 25 (P1) or 21 (P2) days the corresponding peaks of infection (see Methods and Supplementary Note S4). The maximum rate of MIS-C cases for peak P1 corresponds to 0.6 cases per day diagnosed at the Buzzi Children’s Hospital. This value is almost double than that of P2. In contrast, the peak of infection in proximity of P1 is about 5 times smaller than that of P2, hence suggesting a different dynamics of the incidence of infection and MIS-C cases.

### The incidence of MIS-C tends to decrease with time

**Fig. 2:**
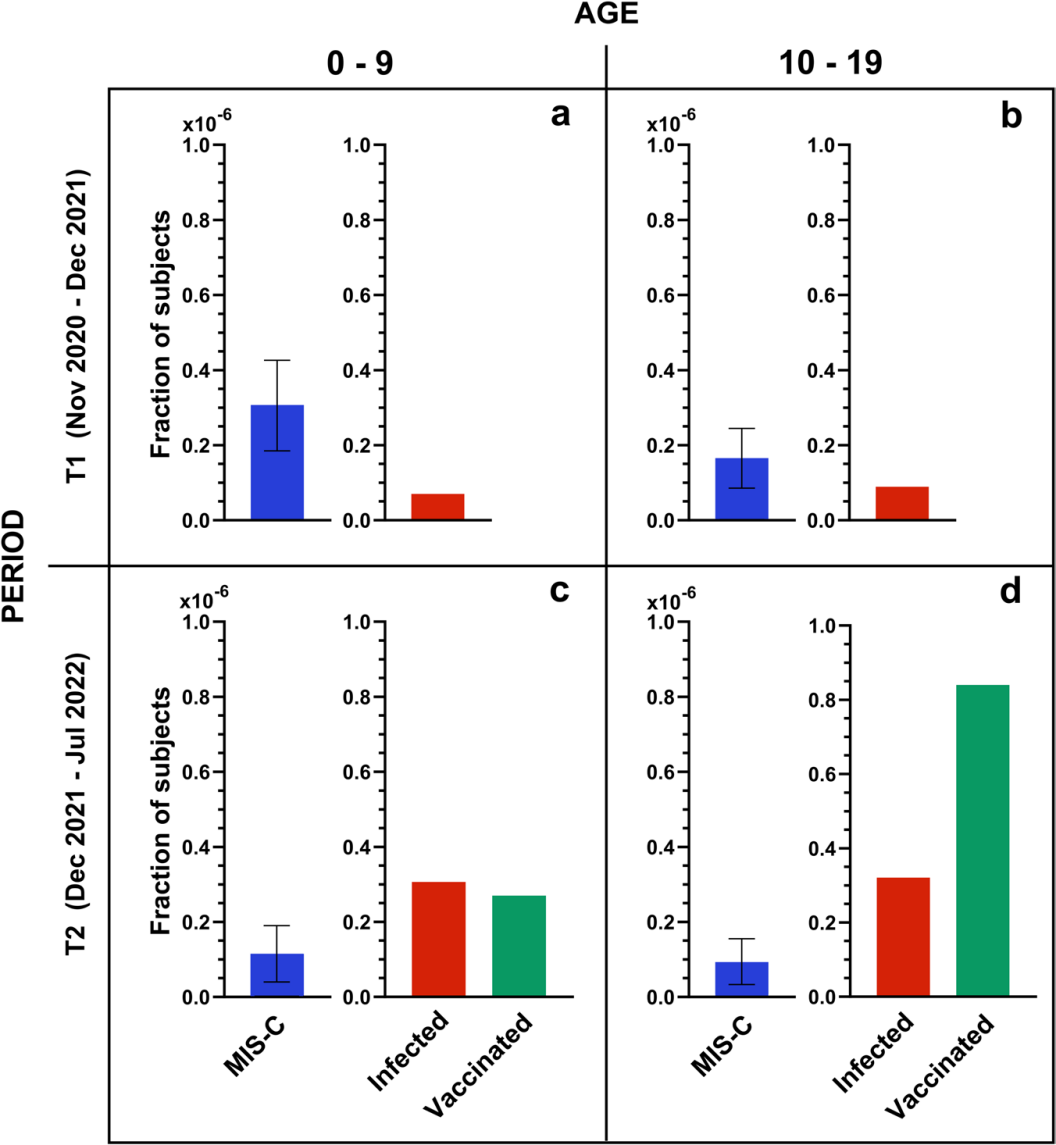
MIS-C patients in relation to the pediatric population. The fraction of MIS-C cases (blue) diagnosed at Pediatric Department of Buzzi Children’s Hospital in Milan, Italy, relative to the population of Lombardy region [25], the fraction of subjects infected by SAR-CoV-2 (red) among the Italian population [24], and the fraction of vaccinated subjects (green) in Lombardy region [25] are reported for the age groups 0–9 (**a, c**) and 10–19 (**b, d**), and for the periods November 9, 2020–December 15, 2021 (**a, b**), and December 16, 2021–July 2, 2022 (**c, d**). The reported values represent the number of subjects divided by the corresponding cohort, i.e. the pediatric population of age 0–9 (**a, c**) and 10–19 (**b, d**) at February 2023 in Italy (red bars) or in Lombardy (blue and green bars). Error bars represent 95% confidence interval (see the “Methods” section). For data of infected and vaccinated subjects, the error bars are smaller than 1% (not displayed). The values of MIS-C cases are multiplied by 10^−6^ as indicated in the axis scale for direct comparison with the other data

In order to investigate the possible causes of the observed decrease of the number of MIS-C cases in the period P2, we considered the effect of the vaccination campaign, which in Italy started on June 3, 2021, for the pediatric population aged 12–15 years, and on December 16, 2021, for the children aged 5–11 years. Accordingly, we considered two age groups, 0–11 and 12–19 years. The fraction of subjects vaccinated with the first dose for these age groups among Lombardy population is shown in Fig. 1c as dark and light violet shadows, respectively. At the time of MIS-C peak P2, the fraction of vaccinated subjects of the 12–19 years group was above 80% and constant, whereas the growth of MIS-C cases at P2 is almost coincident with the increase of the fraction of vaccinated subject of the 0–11-year group, which however reaches a stable value below 30%. Given the different vaccination status of the two age groups, we investigated possible differences in the distribution of MIS-C cases. For direct comparison with the data of the number of infections, we considered two age groups of 0–9 and 10–19 years, and two periods, from November 2020 up to December 15, 2021 (T1), hence before P1 and before the vaccination of the age group 0–11 years, and from December 16, 2021, up to July 2022 (T2). Figure 2 shows that the incidence of MIS-C (blue bar) relative to the incidence of the infection (red bar) is larger in the period T1 (panels a and b) then in T2 (panels c and d), consistently with peaks P1 and P2 in Fig. 1. Overall, the MIS-C incidence is larger for the age group 0–9 (panels a and c), despite the fact that this group shows a slightly lower incidence of infection then the age group 10–19 (panels b and d). For what concerns the vaccinated pediatric population in Lombardy in period T2 (green bar in Fig. 2c, d), the fraction of vaccinated subjects in the age group 10–19 is more than 3 times larger than that of the age group 0–9. Despite this, the MIS-C incidence is only 20% smaller for this age group. Considering that the MIS-C incidence for the age group 10–19 was already smaller before the vaccination campaign (panels a and b), the data under consideration do not allow to correlate the observed decrease of MIS-C cases in period T2 to the number of vaccinated subjects in the pediatric population. Therefore, we also investigated the possible effect of SARS-CoV-2 variants on MIS-C cases.

### The SARS-CoV-2 variant of infection that caused MIS-C can be retrospectively identified by serum antibody fingerprinting

**Fig. 3:**
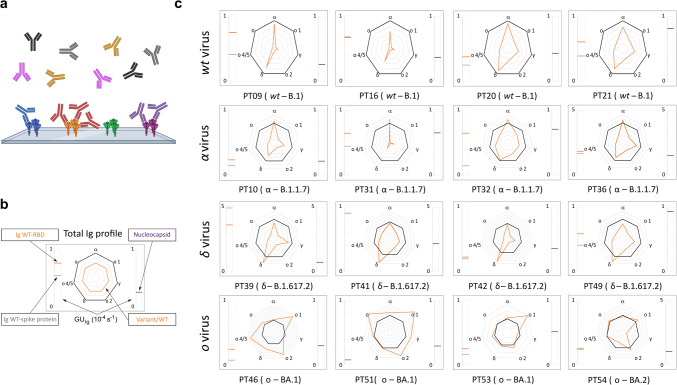
Serum antibody fingerprints of MIS-C subjects against antigens of different SARS-CoV-2 variants. **a** Cartoon of the assay design: Ig antibodies bind the surface-immobilized antigens (i.e., RBD of spike protein or full spike protein). **b** Legend of the fingerprint diagram. The left-side meter reports the quantification of Ig in terms of GU of two WT antigen spots, as indicated. The right-side meter reports the quantification of anti-nucleocapsid antibodies expressed as GU. The radar chart reports the values of RGU for Alpha, Gamma, Delta, and four different Omicron RBD variants. The thick black contour line represents the amount of antibodies binding to WT-RBD taken as reference, hence corresponding to RGU = 1. **c** Selection of Ig fingerprints obtained for sixteen samples of subjects affected by MIS-C and infected with different variants of SARS-CoV-2, as indicated: WT (first row), Alpha (second row), Delta (third row) and Omicron (fourth row)

The variant of a SARS-CoV-2 infection can be retrospectively identified from the repertoire of serum antibody against the different variants of RBD domain of spike protein, as shown in [21, 26]. Accordingly, we analyzed the blood samples that were collected at the time of the hospital admission and then stored for a subset of the children who developed MIS-C. Figure 3 reports a selection of Ig fingerprints in which each box is organized into three parts: the meter on the left-hand side reports the amount of Ig targeting the full spike protein (grey line) and the WT-RBD (orange line) expressed as growth unit (GU) of the biosensor signal; the meter on the right-hand side reports the GU values corresponding to the amount of Ig targeting the nucleocapsid protein; the radar chart in the center reports the RGU profiles computed as the values of GU for different variants (orange line) divided by the GU for WT-RBD taken as reference (black line). As shown in the fingerprints of Fig. 3, the RGU profiles are uncorrelated with the overall amount of Ig against spike protein (left-hand meter) or nucleocapsid (right-hand meter) but provide an independent criterion for the retrospective identification of the variant of infection. In the radar chart, an RGU for a specific variant (orange vertex) larger than the black line reference indicates an Ig amount larger than that of WT-RBD, and this feature is associated to a past infection with that variant of SARS-CoV-2 [21]. Table 2 reports a summary of the variants of infection assigned by serum antibody fingerprinting and of the characteristics of the patients.

**Table 2:** Patients whose variant of infection was assigned by serum antibody fingerprinting

Characteristics	Variant				Vaccinated*
	**WT**	**Alpha**	**Delta**	**Omicron**	
**Fraction of** **tested patients**	9/27	4/6	9/12	5/8	4/4
**Males** (%) **Female** (%)	78 22	100 0	45 55	80 20	50 50
**Age** (years) **avg** **min/max**	6.9 ± 3.0 2/10	8.5 ± 4.4 4/14	7.7 ± 4.0 4/16	10.2 ± 3.9 5/16	10.8 ± 2.1 8/13
**Severity score avg** **min/max**	14.1 ± 3.6 9/21	11.0 ± 1.6 9/13	11 ± 3.3 6/17	12.4 ± 3.0 9/17	13.5 ± 1.3 12/15

*Vaccinated subjects provided serum antibody fingerprints coherent with WT exposure, in agreement with vaccination by WT variant. For these subjects, the variant of infection was assumed to be the variant of prevalence (Omicron)

### The severity of MIS-C is not related to the SARS-CoV-2 variant of infection

**Fig. 4:**
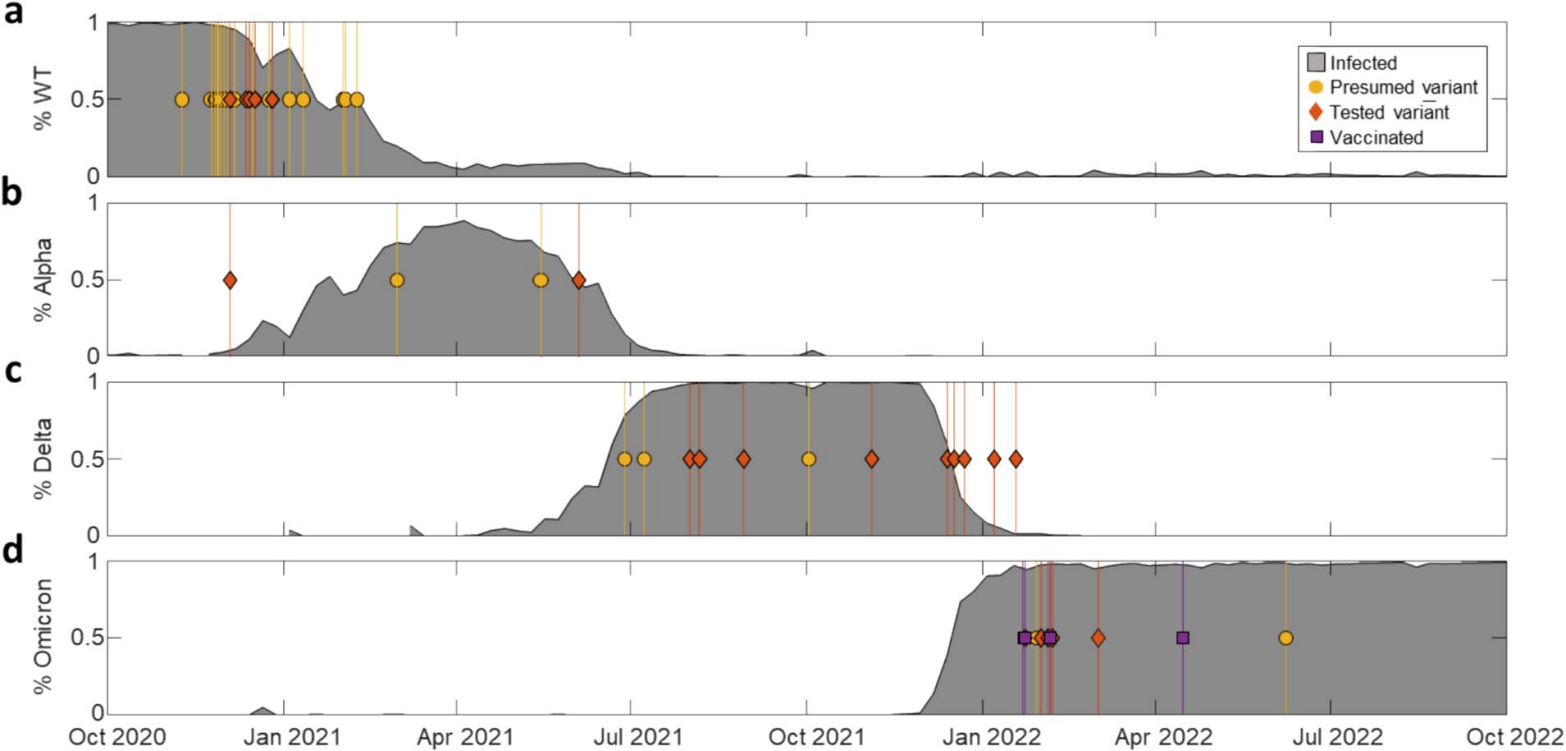
SARS-CoV-2 variants of infection of MIS-C cases. Percentage of infections by WT (**a**), Alpha (**b**), Delta (**c**), or Omicron (**d**) variants in Italy (grey shadow) [27]. Each panel also reports the MIS-C samples whose infection was ascribed to the corresponding variant. The data points and the vertical lines indicate the time of hospital admission of the subjects affected by MIS-C. The retrospective assignment of the variant of infection of MIS-C subjects was based on serum antibody fingerprinting (orange diamonds) or on variant prevalence (more than 50% incidence) at the time of hospital admission (yellow circles). Purple squares in panel d represent patients known to be vaccinated, which resulted as WT profile by serum antibody fingerprint

Figure 4 reports the incidence (grey shadow) for each of the four main variants of the period under study (i.e. WT, Alpha, Delta, and Omicron). Each MIS-C case was ascribed to one of these variants of infection either by serum antibody fingerprinting (orange diamonds) or considering the variant of prevalence (i.e., incidence larger than 50%) at the time of sample collection (yellow circles). Among the 31 samples tested by serum antibody fingerprinting, only 5 were assigned to the variant preceding the variant that was emerging on national scale (Alpha instead of Delta in panel b and Delta instead of Omicron in panel c), and one was assigned to the emerging variant (Alpha instead of WT in panel b). Moreover, only four samples corresponded to subjects vaccinated with the WT variant before MIS-C. The serum antibody fingerprint of these samples was similar to that of WT infection, as expected [21], and the infection causing the MIS-C was ascribed to Omicron variant according to the prevalence (purple squares in Fig. 4d).

**Fig. 5:**
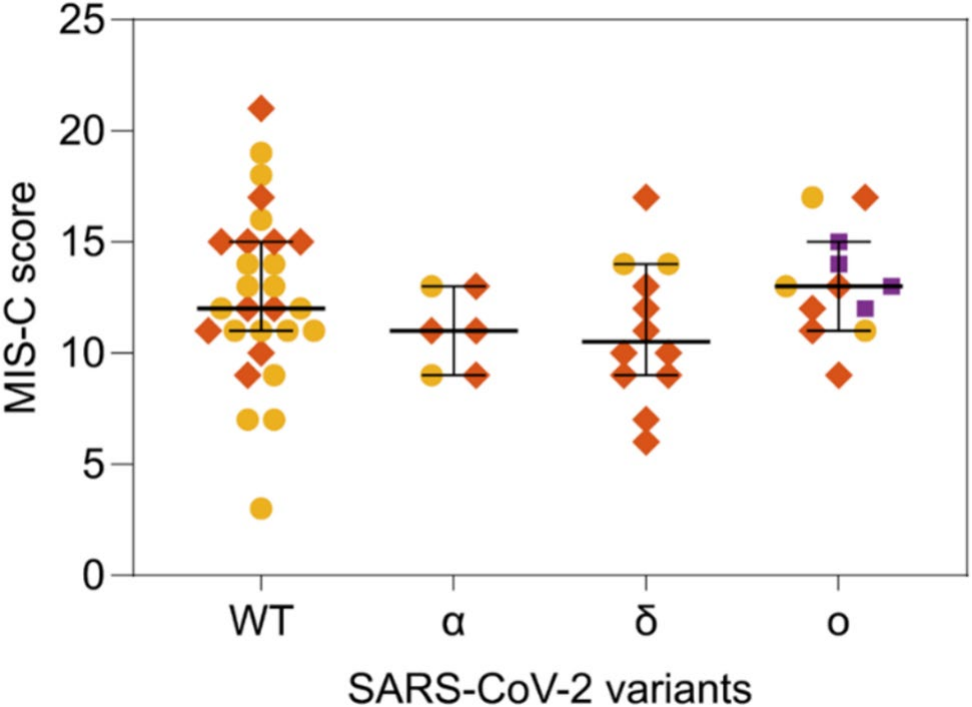
Comparison of the severity score of MIS-C with the SARS-CoV-2 variant of infection. The score assigned to each MIS-C case according to the progression of the disease is reported for the different variants responsible for the infection. For each case, the variant displayed in the abscissa was identified by serum antibody fingerprint (orange diamonds) or as the variant of prevalence at the date of hospitalization (yellow circles). Purple squares represent MIS-C subjects vaccinated before the infection. For each variant, the median and the 95% confidence interval are indicated by horizontal bars

The retrospective identification of the variant of infection enables to investigate the dependence of the severity of MIS-C on SARS-CoV-2 variants. Figure 5 reports the severity score assigned to each MIS-C case for the different variants of infection. Despite a larger distribution of score values of the WT variant, which is ascribed to the larger number of cases, both the median values and confidence intervals of the score (as well as the average and the standard deviation) are similar for all the variants. As also confirmed by analysis of variance (see the “Methods” section and Supplementary Note S5), the distributions of severity score values reported in Fig. 5 do not show significant dependences on the variant of infections.

## Discussion

The World Health Organization (WHO) declared COVID-19 a pandemic in March 2020 [28]. In the spring of 2020, a significant increase in Kawasaki disease-like syndrome among children was reported, leading to a multisystem inflammatory condition known as MIS-C [29–32]. MIS-C has been shown to be a potentially life-threatening illness, characterized by severe and aberrant systemic inflammation leading to nonspecific symptoms, such as gastrointestinal, cardiac, respiratory, hematological, and neurological disorders [15]. The incidence rates of MIS-C vary across different geographic regions. Increased case numbers have been observed in Europe, the Americas, Africa, South Asia, and the Middle East, while East Asian countries report significantly fewer cases. This geographic distribution of MIS-C incidence is likely influenced by a combination of environmental factors, social determinants of health disparities, and genetic backgrounds [32, 33].

A wide clinical spectrum of MIS-C presentations has been described. In our study, we proposed a severity score designed to assign a graded evaluation for each affected organ, based on both clinical and biochemical parameters. Other classifications have been reported in the literature aiming to stratify MIS-C severity [34, 35]. Notably, Rao et al. [36] applied heterogeneity-adaptive latent class analysis and identified three distinct clinical profiles: Class 1, comprising nearly half of the patients, was associated with the most severe manifestations, including frequent ICU admissions, elevated inflammatory markers, and significant involvement of the cardiac, respiratory, and renal systems. Class 2 represented a moderate presentation, with multi-organ involvement and features overlapping with acute COVID-19, while Class 3 reflected milder cases. Similarly, Ma et al. [35] identified three clusters, distinguished by predominant respiratory symptoms (8.0%), cardiac complications and shock (37.6%), and clinically mild, undifferentiated cases (54.5%). All these approaches aim to stratify MIS-C patients based on clinical presentation and severity. However, while our proposed score quantitatively grades severity using more specific clinical and laboratory parameters, the other classifications group patients into phenotypic subtypes without assigning a numeric score. These complementary methods enhance our understanding of the clinical heterogeneity of MIS-C and may support more tailored patient management and treatment strategies.

A global decline in MIS-C cases has been observed alongside the evolution of the SARS-CoV-2 virus [37]. US national surveillance data showed that the proportion of total MIS-C cases decreased over time with sequential SARS-CoV-2 variants, with 58.0% occurring pre-Delta and 16.1% occurring during the Omicron predominant phase [35]. U.K. have likewise demonstrated a significant decline in MIS-C incidence during the Omicron-predominant period, both in terms of absolute case numbers and clinical severity [38]. Similarly, research from Asian countries such as South Korea [12] and Japan [39] showed a declining trend in MIS-C cases during the Omicron wave. Accordingly, MIS-C is generally perceived as a less threatening disease in the latest stage of the pandemic, but this can be ascribed to the gained experience and preparedness of medical centers enabling more rapid and effective treatments. Specifically, it was reported that Omicron-related MIS-C cases have milder symptoms and organ damage than previous variants, even though the variant itself is more transmissible [40]. As reported, the relative proportion of children in the respiratory as well as shock and cardiac clusters gradually decreased after the emergence of the Omicron variant in the USA, with the more mild, undifferentiated cluster predominating [35]. Studies in the UK reported a marked reduction in hospitalizations and ICU admissions for MIS-C post-Omicron emergence, with milder phenotypes predominating [41]. In Asian countries a shift toward less severe clinical presentations was also reported [12, 39].

In order to unveil hidden correlations of MIS-C dynamics with the pandemic evolution, we analyzed in detail the MIS-C cases treated at the main pediatric hospital of the city of Milan (Italy), a reference center for pediatric patients with complex conditions in the whole Lombardy region, an area with a population of nearly 10 million inhabitants. The results confirm a decrease in the incidence of MIS-C in relation to infections. Conversely, our data also support the notion that the severity of MIS-C in hospitalized patients remains largely consistent across different viral variants, including Omicron.

As shown in Fig. 1, the number of MIS-C cases tends to follow the infections up to a certain extent. The fact that the MIS-C peak P2 in Fig. 1 is smaller than P1, despite a larger peak of infections, can be ascribed either to the decrease of the susceptible population, due to vaccination and previous infections, or to the characteristics of the virus variants.

In the early phase of the pandemic, the incidence of MIS-C among unvaccinated subjects was estimated as 300 per million SARS-CoV-2 infections in persons younger than 21 years [42]. A decrease in the incidence of MIS-C during the Delta and the Omicron waves and a protective effect due to vaccination has been shown [43], down to 0.6 cases per million among vaccinated children [44]. In our population, comparing different pediatric age groups (Fig. 2), which had a different vaccination history, we could not derive a direct protective effect of vaccination against MIS-C. Moreover, among the 12 MIS-C cases ascribed to Omicron infection, as much as 4 were vaccinated, although with the first vaccine version against WT strain. However, the overall reduction of infections due to the vaccination campaign clearly yielded also a reduction of MIS-C cases down to rare events.

As highlighted by Castaldo et al. [33], various factors could potentially explain the observed changes in the incidence and severity of MIS-C. These include alterations in the human immune response, the gradual development of immunological memory to SARS-CoV-2 over time, mutations in critical amino acids of the S protein in VOCs, and the interplay between the immune response triggered by vaccination and the reduced neutralization efficacy of vaccines against VOCs.

In contrast, the factors that could affect the severity of MIS-C are unknown [45, 46]. According to the data collected in this study, the severity of MIS-C does not show clear trends with age or sex of the children, or virus variant, despite the decrease of the number of cases. In particular, we assigned each MIS-C case to a variant of infection according to serum antibody fingerprinting and data on the variant on prevalence in the period of the hospitalization of the children. The analysis of serum antibody repertoire confirmed the infections due to largely prevalent variants, but also enabled a correct assignment in case of coexistence of different variants (Fig. 4). As expected, the MIS-C peak P1 in Fig. 1c is all due to the WT variant, whereas the peak P2 can be predominantly ascribed to the Omicron variant. Given the smaller incidence of MIS-C in P2 relative to the infected population (Figs. 1 and 2), we cannot rule out a smaller tendency of the Omicron variant to induce MIS-C. In contrast, the severity of MIS-C does not show a decreasing trend with time and hence with the variant progression (Fig. 5). Therefore, if the overall decrease of the number of MIS-C cases cannot be decoupled from the progression of the SARS-CoV-2 variants, because of the concomitant decrease of the susceptible population, the severity of MIS-C seems unaffected and rather stable across the different variants of infection. Thus, once initiated, the course of MIS-C appears to be independent of the characteristics of the triggering variants, although later variants may be less likely to induce MIS-C.

When conducting this research, it is important to consider potential limitations. Firstly, the small sample size restricts the robustness of the analysis; thus, studies on larger cohorts are needed to expand the sample size and validate these findings. Secondly, MIS-C cases are from a single center, which may limit the generalizability of our results, as variations in patient demographics, clinical practices, and institutional protocols across different centers could influence outcomes. Furthermore, there is considerable variability among the participating populations, including factors such as age and vaccination status. Additionally, another limitation of our study is related to the severity scale employed. Currently, there are no globally validated severity scales for MIS-C; therefore, caution should be exercised in interpreting our findings, as the scale we used has been validated only within a specific pediatric population. Future studies utilizing cluster analysis could help refine surveillance case definitions and more effectively identify patient groups at higher risk for severe outcomes. Finally, the identification of the SARS-CoV-2 variant was conducted retrospectively.

In conclusion, our results clearly indicate a decline in the incidence of MIS-C associated with COVID-19 infections. The potential role of a combination of factors in the reduction of MIS-C incidence over time cannot be excluded, even though precisely quantifying the contribution of each factor is challenging. However, our results also reinforce the idea that the severity of MIS-C in hospitalized pediatric patients has remained largely consistent across various virus variants. Once MIS-C is initiated, its progression appears to be independent of the specific characteristics of the triggering variants.

## Supplementary Information

Below is the link to the electronic supplementary material.

ESM 1 (PDF 265 KB)

## Data Availability

Data and data sources are provided within the manuscript.
