# Supplementary material for: Dynamics of Multisystem Inflammatory Syndrome in Children (MIS-C) associated to COVID-19: steady severity despite declining cases and new SARS-CoV-2 variants—a single-center cohort study
Source: Eur J Pediatr. 2025 May 7;184(6):327. doi: 10.1007/s00431-025-06153-1 (PMC12058826; doi:10.1007/s00431-025-06153-1)
Supplement: Supplementary file 1 — (PDF 265 KB) [file 431_2025_6153_MOESM1_ESM.pdf]

Dynamics of Multisystem Inflammatory Syndrome in Children (MIS-C) associated to COVID-19: Steady Severity Despite Declining Cases and New SARS-CoV-2 Variants. A Single-Center Cohort Study.

Supplementary Material

S1. Severity score

To assess the severity of multisystem involvement, we adopted a scoring approach assigning specific scores to various affected domains. These domains include renal, cardiac, gastrointestinal, neurological, pulmonary, dermatological/mucosal, endocrine, metabolic, and electrolyte disturbances. As summarized in Table S1, each domain is scored individually, ranging from 0 to 2 points depending on the observed severity. By summing the scores from each domain, a cumulative severity score is calculated, providing an overall measure of the severity of patient's clinical condition. Additionally, the table includes important clinical outcomes, such as the requirement for intensive care unit admission during initial hospitalization, hospitalization duration and presence of fever.

Table S1. Definitions of severity sub-scores and variables related to hospitalization.

ORGAN	SCORE		
	**0**	**1**	2
Kidney [1]	Normal creatinine	Increase in creatinine < 50%	Increase in creatinine > 50%
Heart [2]	Ejection fraction > 45%	Ejection fraction ≤ 45%	Ejection fraction ≤ 35%
Gastrointestinal system [3]	None	Abdominal symptoms	Abdominal symptoms and ultrasound pathological signs eco
Central nervous system	None or nonspecific neurological signs	mild encephalopathy<br>+ focal neurological signs<br>+/- focal EEG abnormalities	encephalopathy<br>+ focal neurological signs<br>+ diffuse EEG abnormalities
Lung	None	respiratory symptoms and/or O2 therapy and/or imaging alteration	Ventilation need
Fever [4]	< 3 days	3-7 days	> 7 days
Skin/mucosal involvement	None	skin	Skin and mucosal
Endocrine system	None	< 2 hormonal alterations (including thyroid and/or adrenal hormones and/or insulin)	≥ 2 hormonal alterations
Metabolic involvement	None	< 2 metabolic parameters (including increased FBG, dyslipidemia, insulin resistance, hepatic alteration)	≥ 2 metabolic parameters
Duration of hospitalization in pediatric intensive care unit	None	≤ 3 days	> 3 days
Hospitalization	< 10 days	11-14 days	≥ 15 days
Weight loss	None	≤ 0.5 BMI-SD	> 0.5 BMI-SD
Electrolyte imbalance	None	1 (sodium or potassium)	2 altered electrolytes

S2. Assessment of the influence of age of subjects on the severity score

The possible influence of the age of the subjects on the severity score of MIS-C was assessed by analyzing the dependence between these two parameters. Figure S1 shows a scatter plot of all MIS-C score data as a function of the age of children. From the linear regression of these data, we obtained a negligible slope and a Pearson correlation coefficient of 0.0258, hence supporting the notion of no significant dependence of the score on the age of subjects. The parameters of the analysis are reported in the figure caption. Very similar results were obtained also using a Welch's t-test, which does not assume equal variances.

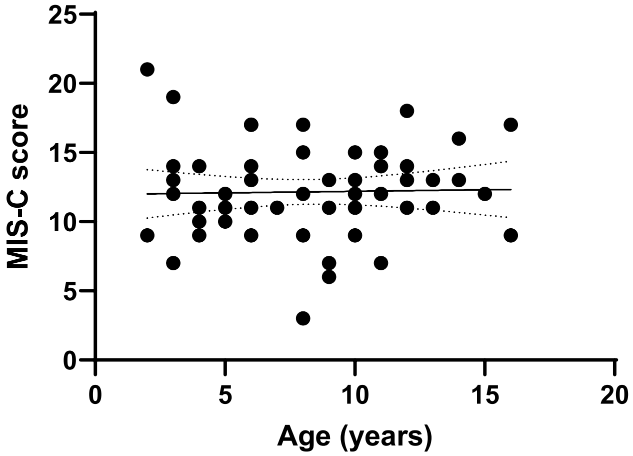

Figure S1. Severity score of MIS-C as a function of subject's age. The continuous line represents a linear fit with slope 0.0228 and intercept 11.97. The dotted lines represent the 95% confidence interval of the fit. The goodness of the fit is very low (R^2 = 0.00067) and the slope is not significantly different from zero (P value of 0.849). The Pearson correlation coefficient is 0.0258.

S3. Assessment of the influence of sex of subjects on the severity score

The possible influence of children's sex on the severity score of MIS-C was assessed using Student's t-test. Figure S2 shows the distributions of score values for males and females. We conducted an unpaired Student's t-test on these two groups of data, and the results of the analysis indicated that there is no significant difference. The parameters of the analysis are reported in Table S2.

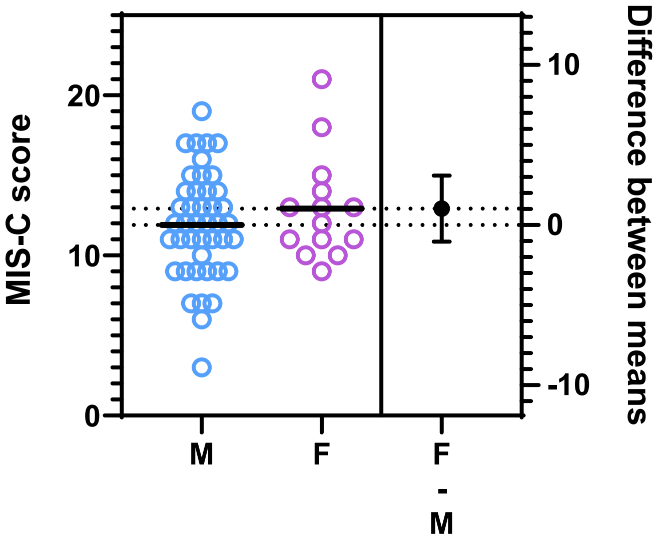

Figure S2. Severity score of MIS-C for males and females. The left panel reports the distributions of score values for males (M) and females (F). The horizontal black segments and the dashed lines represent the mean value of each distribution of scores. The right panel reports the difference between the means. The error bars represent the 95% confidence interval.

Table S2. Parameters of unpaired Student's t-test performed on the data of Figure S2.

P value of unpaired t-test	0.3234
Significantly different (P < 0.05)?	No
One- or two-tailed P value?	Two-tailed
t, df	t=0.9965, df=55
Mean of males score	11.91
Mean of females score	12.93
Difference between means ± SEM	1.022 ± 1.025
95% confidence interval	-1.033 to 3.076
R squared (eta squared)	0.01773
F, DFn, Dfd	1.031, 42, 13
P value of F test to compare the variances	0.9910

S4. Latency between MIS-C cases and COVID-19 infections

The latency between MIS-C cases and COVID-19 infections was obtained from the time delay to be applied to the data of the number of infections per day in order to yield the best linear regression with the data of the number of MIS-C cases per day. Figure S3 reports the goodness of such linear regressions expressed in terms of the coefficient of determination R^2, using the data of infections per day in the Lombardy region shifted by different time delays. We considered only the MIS-C data around the two main peaks of cases: P1 from October 25, 2020 to March 17, 2021, and P2, from December 8, 2021 to February 26, 2022. From parabolic fits of the R^2 data in Figure S3 we obtained latencies of 25 days for P1 and 21 days for P2. The MIS-C and infections data and the corresponding linear fits for these optimal time delays are reported in Figure S4. The comparison of the slopes of the linear fits shows that the incidence of MIS-C cases computed over the population of Lombardy region is about a factor of ten smaller for P2 relative to P1, as also shown in Figure 1.

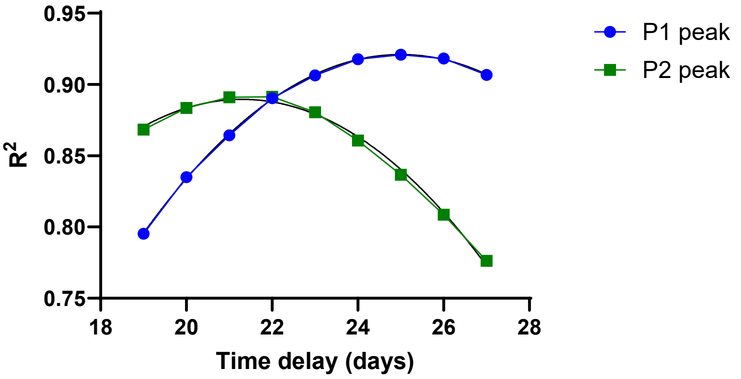

Figure S3. Goodness of linear regression of MIC-C cases as a function of infections with different time delays. The coefficient of determination R^2 obtained from linear fits of the number of MIS-C cases per day as a function of the number of COVID-19 infections per day in Lombardy region is reported for different time delays applied to the data of infections. The fits were limited to the periods around the peaks P1 (blue) and P2 (green) of MIS-C cases. The black curves are quadratic fits yielding maximum values of R^2 at 25 days for P1 data and 21 days for P2 data.

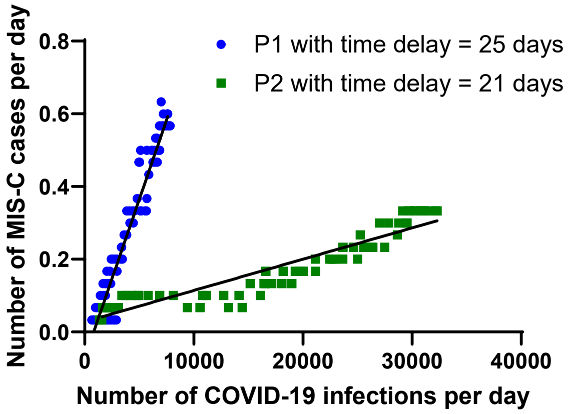

Figure S4. Linear dependence of the number of MIS-C cases per day as a function of the number of COVID-19 infections per day in Lombardy region considering the latency. The data for MIS-C peak P1 (blue) and P2 (green) are plotted as a function of the infections with a time delay of 25 days for P1 and 21 days for P2, representing the latency obtained from the analysis reported in Figure S3. The black lines represent linear fits yielding slopes of 8.7 and 0.86 MIS-C cases per 100.000 infections for P1 and P2, respectively

S5. Comparison among severity scores of MIS-C due to different variants

The severity score of MIS-C for different SARS-CoV-2 variants that caused the infections showed similar distributions of values, as reported in Figure 5. To accurately test the possible differences among these distributions of scores we performed a one-way analysis of variance (ANOVA) with Tukey's post hoc analysis. The mean values and the 95% confidence interval of the distributions are shown in Figure S5, and the differences between the mean values are reported in Figure S6. The parameters obtained using ANOVA test are reported in Table S3, while Table S4 reports the parameters obtained from Tukey's comparisons test. The analysis indicates that there is no significant difference among the score distributions for these variants.

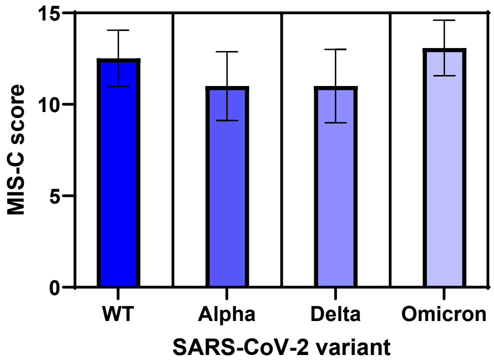

Figure S5. Mean values and confidence intervals of the distributions of MIS-C severity score for the considered SARS-CoV-2 variants.

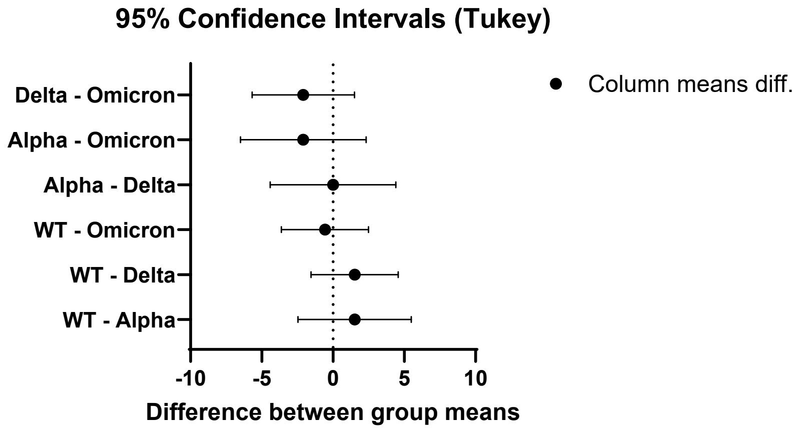

Figure S6. Differences between mean values and confidence intervals obtained from the comparison of the distributions of MIS-C severity score for the SARS-CoV-2 variants considered. The parameters are obtained with Graphpad Prism version 10 using ANOVA test with Tukey's post hoc analysis.

Table S3. Summary of parameters of ANOVA test.

F	1.148
P value	0.3384
Significant diff. among means (P < 0.05)?	No
R squared	0.06101
Null H. Probability of all population means identical	84.33%
Alternative H: Probability of distinct population means	15.67%

Table S4. Summary of parameters of Tukey's multiple comparisons test.

Comparisons test	Mean Diff.	95% CI of diff.	Adjusted P Value
WT vs. Alpha	1.519	-2.454 to 5.491	0.7421
WT vs. Delta	1.519	-1.535 to 4.572	0.5552
WT vs. Omicron	-0.5648	-3.619 to 2.489	0.9609
Alpha vs. Delta	0.000	-4.401 to 4.401	>0.9999
Alpha vs. Omicron	-2.083	-6.484 to 2.318	0.5948
Delta vs. Omicron	-2.083	-5.677 to 1.510	0.4227

References

1. Makris K, Spanou L (2016) Acute Kidney Injury: Definition, Pathophysiology and Clinical Phenotypes. Clin Biochem Rev 37(2):85–98.
2. Hudson S, Pettit S (2020) What is ‘normal’ left ventricular ejection fraction? Heart 106(18):1445–1446. https://doi.org/10.1136/heartjnl-2020-317604
3. Cattalini M, Taddio A, Bracaglia C, et al. (2021) Childhood multisystem inflammatory syndrome associated with COVID-19 (MIS-C): a diagnostic and treatment guidance from the Rheumatology Study Group of the Italian Society of Pediatrics. Ital J Pediatr 47(1):24. https://doi.org/10.1186/s13052-021-00980-2
4. Yan F, Pan B, Sun H, Tian J, Li M (2019) Risk Factors of Coronary Artery Abnormality in Children With Kawasaki Disease: A Systematic Review and Meta-Analysis. Front Pediatr 7. https://doi.org/10.3389/fped.2019.00374
